# Experience of Vancomycin Therapeutic Drug Monitoring in Two Multidisciplinary Hospitals in Latvia

**DOI:** 10.3390/medicina58030370

**Published:** 2022-03-02

**Authors:** Inga Mauliņa, Karīna Darbiniece, Lāsma Miķelsone-Jansone, Renārs Erts, Dace Bandere, Angelika Krūmiņa

**Affiliations:** 1 Riga East Clinical University Hospital, Hipokrata St. 2, LV-1038 Riga, Latvia; 2 Vidzeme Hospital, Jumaras St. 195, LV-4201 Valmiera, Latvia; 3 Department of Pharmaceutical Chemistry, Faculty of Pharmacy, Riga Stradins University, LV-1007 Riga, Latvia; dace.bandere@rsu.lv; 4 Pauls Stradins Clinical University Hospital, Pilsonu St. 13, LV-1002 Riga, Latvia; karinaevalde@inbox.lv; 5 Faculty of Medicine, Riga Stradins University, 16 Dzirciema St., LV-1007 Riga, Latvia; lasmamikelsone21@gmail.com; 6 Faculty of Medicine, University of Latvia, Raina Blvd. 19, LV-1586 Riga, Latvia; renars.erts@lu.lv; 7 Baltic Biomaterials Centre of Excellence, Headquarters at Riga Technical University, Dzirciema Street 16, LV-1007 Riga, Latvia; 8 Department of Infectiology, Riga Stradins University, 16 Dzirciema St., LV-1007 Riga, Latvia; angelika.krumina@rsu.lv

**Keywords:** antimicrobial resistance, clinical pharmacist, intermittent vancomycin administration, drug monitoring protocol, vancomycin concentration

## Abstract

*Background and Objectives*: Management of infectious diseases is a huge burden to every healthcare system worldwide. Antimicrobial resistance, including antibacterial resistance, is an increasing problem worldwide; therefore, more new antibiotics are necessary to be discovered. Meanwhile, “old” antibacterial agents are still administered to fight infectious diseases caused by resistant bacteria. One of these antibacterial agents is vancomycin, which is effective in treating serious systemic infections caused by gram-positive bacteria. Thus, it is necessary to perform vancomycin concentration measurements in plasma due to its narrow therapeutic index. Various approaches are implemented for more precise therapy, including therapeutic drug monitoring (TDM) of vancomycin and with a supervision of a clinical pharmacist. The purpose of the study was to investigate if the TDM practice is improved with a local vancomycin TDM protocol applied in a hospital. The results of TDM in two multidisciplinary hospitals, one with a local TDM protocol implemented and applied and the other with no local TDM protocol implemented and applied, were compared. *Materials and Methods*: A retrospective study was performed in two multidisciplinary hospitals in Latvia. The data were collected for a time period of 4 years (2016–2020) in a hospital without a local TDM protocol and for a time period of 2 years (2018–2020) in a hospital with a local TDM protocol, starting with a period of time when the vancomycin TDM protocol was developed. The data about the patients included in the study were analyzed based on gender, age, body weight, and renal function. Vancomycin therapy was analyzed based on dosing schemes (vancomycin dose and dosing interval), data about loading and maintenance doses, vancomycin concentration, and details about vancomycin concentration (sampling time and concentration level). *Results:* Differences between the hospitals were found in terms of the initiation of vancomycin administration and concentration sampling. In the hospital with a TDM protocol compared with the hospital without a TDM protocol, more accurate initiation was found, alongside adaption of therapy (97.22% vs. 18.95%, *p* < 0.001), better performance of administration of a loading dose (22.73% vs. 1.29%, *p* < 0.01), and reaching of target concentration (55.56% vs. 35.29%, *p* < 0.01). Concentration sampling in the correct timeframe before the vancomycin dose and vancomycin administration did not show statistically better results in either of the hospitals (4.60% vs. 6.29%, *p* = 0.786). *Conclusions*: Better results of adequate adjustments of vancomycin therapy were achieved in the hospital with a TDM protocol. In the long term, sustainable results and regular medical professionals’ training is necessary.

## 1. Introduction

Antimicrobial resistance is an increasing problem worldwide; therefore, it is necessary to discover new antibiotics. Meanwhile, “old” antibacterial agents are still administered to fight infectious diseases caused by resistant bacteria. One of these antibacterial agents is vancomycin. It is effective in treating serious systemic infections caused by gram-positive bacteria. Vancomycin is a glycopeptide antibiotic that acts against a variety of Gram-positive bacteria, including methicillin-resistant Staphylococcus aureus. It was discovered in the 1950s. At that time, vancomycin utilization was discontinued due to significant impurity issues in the formulation. In 1980, the administration of vancomycin was revived in clinical practice [1].

To treat various systemic infectious diseases, vancomycin should be administered intravenously. It is a narrow-spectrum antibiotic whose subtherapeutic concentrations (i.e., below 10 mg/L) are associated with an increased risk of developing antibiotic resistance, while supratherapeutic levels (above 20 mg/L) may contribute to the potential toxicity, including nephrotoxicity [2]. The pharmacokinetic profile of intravenous vancomycin is very complex and can vary extensively among different patient groups, depending on age, weight, co-morbidities, and renal function. Therefore, individual dose adjustment and monitoring of vancomycin trough concentrations are recommended [3].

Therapeutic drug monitoring (TDM) should be performed to establish whether an optimal therapeutic vancomycin concentration is reached, and appropriate therapy modification is performed as a result. Vancomycin TDM is an important part of a management strategy of vancomycin therapy [4]. There are studies confirming that utilization of vancomycin TDM allows therapeutic concentrations to be reached more rapidly, thus possibly allowing to shorten vancomycin therapy and therefore reduce the length of patient hospitalization [5]. To promote safe and successful vancomycin therapy, the first consensus guidelines for therapeutic monitoring of vancomycin in adults were published in the USA in 2009. The guidelines outlined several important aspects, including serum concentration targets, analysis, and dosing to standardize vancomycin therapy [6].

Over time, to improve vancomycin TDM, various medical organizations and competent authorities have also developed clinical practice guidelines [7,8]. However, despite efforts, proper adaptation of vancomycin therapy, including adequate TDM, remains a major challenge of successful treatment in many hospitals. Recent studies suggest that the number of correct vancomycin TDM in clinical practice is lower than expected and it may contribute to the increased resistance against vancomycin [4].

Unfortunately, there is a limited number of recommendations stating the appropriate improvement process of vancomycin TDM in routine clinical practice. Furthermore, there are scientific reports suggesting that the knowledge of vancomycin TDM practice is not sufficient among medical professionals [9]. To improve TDM practice, various methods, such as an inclusion of a clinical pharmacist in a multidisciplinary team, targeted educational programs, and standardized vancomycin therapy protocols, have been implemented [3,10,11].

The purpose of this study was to investigate if TDM practice improves with a local vancomycin TDM protocol applied in a hospital. The results of TDM in two multidisciplinary hospitals—one with a local TDM protocol implemented and applied and the other with no local TDM protocol implemented and applied—were compared. Such a study comparing TDM practices is very rare and therefore it should provide additional evidence-based results for improving TDM practices worldwide.

## 2. Materials and Methods

A retrospective study was performed in two multidisciplinary hospitals in Latvia: in a hospital with a vancomycin TDM protocol and in a hospital without a vancomycin TDM protocol.

The data were collected for a time period of 4 years (2016–2020) in the hospital without a TDM protocol and for a time period of 2 years (2018–2020) in the hospital with a TDM protocol, starting from the time when the vancomycin TDM protocol was developed.

In the hospital with a TDM protocol, the protocol was developed by a clinical pharmacist for patients with normal or impaired renal function and for patients with renal replacement therapy. The protocol includes the instructions for administration of vancomycin, including the dosing and intervals, as well as monitoring vancomycin concentration in plasma.

The loading dose is calculated based on the body weight, which is recommended for an initiation of vancomycin therapy, followed by maintenance doses dependent on renal function. The first TDM of vancomycin in patients with normal renal function is recommended before the third or fourth dose. Trough levels should be obtained immediately before the next scheduled dose (within 30 min). The target range for vancomycin trough levels is 15–20 mg/L, but it needs to be at least 10 mg/L for all infections to avoid the potential of resistance. Frequency of TDM depends on the concentration of vancomycin and stability of renal function [5].

It should be noted that in the hospital with a TDM protocol, before the vancomycin TDM protocol, the reference interval for vancomycin trough levels was expressed as 3.5–6.9 µmol/L (equal to 5–10 mg/L). After the implementation of the protocol in 2018, the reference range was changed and expressed as 15–20 mg/L according to the international guidelines. In the hospital without a TDM protocol, none of the reviewed laboratory test results indicated a reference interval for vancomycin concentration.

The study was approved in the Ethical Committee of Riga Stradins University, 16 Dzirciema Str., LV-1007, Riga, Latvia, approval no. 6-1/07/60, 25 June 2020.

The selection of medical data was performed through the database containing data of performed vancomycin concentration measurements assigned by The National Health Service of Latvia. Exclusion criteria were as follows: patients under 18 years of age; patients on renal replacement therapy; vancomycin therapy initiated several times during the same episode of hospitalization; TDM performed during peroral vancomycin therapy, TDM performed without initiated vancomycin therapy; patients with any missing information about the actual result of vancomycin concentration. In total, 126 patients from the hospital without a TDM protocol and 44 patients from the hospital with a TDM protocol were included in this study.

Data about the patients were analyzed based on gender, age, body weight, and renal function. Data about vancomycin therapy were analyzed according to dosing schemes (vancomycin dosage and dosing interval), data about loading and maintenance doses, vancomycin concentration, and details about vancomycin concentration (sampling time of blood and concentration level in plasma).

A 95% confidence interval and a 10% margin of error were chosen for this study. Taking into account the total number of vancomycin recipients, the hospital without a TDM protocol had an estimated sample size of 126. The hospital with a TDM protocol had an estimated sample size of 44 based on the above parameters.

Statistical analysis was performed using R Core Team (2020), IBM SPSS Statistics (2020), and Microsoft Excel 2013. Various analysis parameters, central trend indicators, percentages, and minimum and maximum values were evaluated. For data that did not comply with normalization, the median (Me) and the interquartile ratio (IQR) were used for the quantitative assessment. If data were normally distributed, then averages (M) and standard deviation (SD) were used. The independent-samples T test was used to compare mean data between groups if data were within the normal distribution, and the Mann-Whitney U test was used if data were not within the normal distribution. For the processing of qualitative data. The Pearson’s chi-squared test and Fisher’s exact statistical test were performed according to the number of expected values (>5 or <5, respectively). For correlation analysis, the Pearson or Spearman test was performed. Data were considered statistically significant if the *p*-value was less than 0.05.

## 3. Results

In total, 126 patients in the hospital without a TDM protocol and 44 patients in the hospital with a TDM protocol were included in the statistical analysis. The demographic and clinical characteristics of the study patients are shown in Table 1. The groups were similar in terms of gender, weight, and renal function, but not in age or length of hospitalization. In the hospital without a TDM protocol, a wider age range was observed and the average length of hospitalization was longer.

It has been documented that two patients (1.59%) received the loading dose in the hospital without a TDM protocol and 10 patients (22.73%) received the loading dose in the hospital with a TDM protocol (*p* < 0.01).

Elimination of vancomycin is almost exclusively renal, which is why vancomycin maintenance doses depend on renal function, usually evaluating GFR. To evaluate the reasoning of a maintenance dosing regimen based on renal function, patients enrolled in the study were divided into six GFR groups (Table 2). The Fisher’s exact statistical test was performed to compare the association of GFR groups between the hospital with a TDM protocol and the hospital without a TDM protocol, and the difference was not statistically significant (*p* = 0.636).

It is important to analyze renal function, as it directly affects the vancomycin level in plasma and, subsequently, the outcome of vancomycin therapy, especially in patients with augmented renal function or severe renal impairment. The dosing regimen in both hospitals depending on the GFRin percentage is shown in Table 3 and Table 4.

In the hospital without a TDM protocol (Table 3), the most common dosing regimen for GFR groups 1, 2, and 3 was 1000 mg vancomycin every 12 h. In groups with impaired renal function, GFR < 44 mL/min/1.73 m^2^, (GFR groups 4, 5, and 6), the most common dosing regimen was 1000 mg of vancomycin every 24 h (1 g q24h). More variations in treatment regiments appeared in groups GFR 1 and GFR 2. In most of the cases, patients received 1000 mg vancomycin every 12 h. But there were also treatment schemes that included both prolongation (1000 mg every 24 h) or shortening (1000 mg every 8 h) of a dosing interval.

In the hospital with a TDM protocol (Table 4), the most common dosing regimen was 1000 mg vancomycin every 12 h in all GFR groups, except group GFR 4, where all patients received 1000 mg vancomycin every 24 h. In this hospital, patients from group GFR 1 were more likely to receive vancomycin dosing regiments of 1000 mg every 8 h or 1500 mg every 12 h. It should be added that such regiments were not applied in the hospital without a TDM protocol. In groups with a lower GFR, the reduction of a maintenance vancomycin dose (500 g every 12 h) or the prolongation of an interval between doses (1000 mg every 24 h) were applied as therapeutic strategies.

For the correct interpretation of the results of vancomycin concentration, the correct timing of taking a blood sample should be ensured and aligned with the administration of vancomycin dose. It should be noted that, in this study, we established that the correct timing of taking a blood sample would be right before, but not earlier than, 30 min before the administration of a vancomycin dose.

From all the blood samples drawn in the hospital without a TDM protocol, only for 87 (19.95%) out of 459 concentration samples, there were data about both blood sampling and vancomycin administration time. In contrast, in the hospital with a TDM protocol, all necessary data was documented in 175 (97.22%) out of 180 concentration measurements.

Only cases with both sampling and administration times were taken for the analysis of the time frame between the blood samples and administration of the next vancomycin dose (87 vs. 175 concentration measurements, respectively) (Table 5).

In the hospital without a TDM protocol, most of the cases of blood samples of vancomycin concentration were drawn within 1–3 h prior to the next dose (34.48%) in most of the cases, whereas in the hospital with a TDM protocol, they were drawn within 2–3 h prior to the next dose (21.71%) or up to 3 h after administration of vancomycin (20.00%). Both sampling and administration times in medical histories were dated as done at the same time in 12% and 4.60% of cases in the hospitals with a TDM protocol and without a TDM protocol, respectively. As it was not possible to determine whether the analysis was taken immediately before or after the dose, based only on vancomycin concentration level, these cases were separated. For example, the time of both manipulations was recorded at 6:45 but vancomycin concentration was 33.8 mg/L or the recorded time was at 08:00, but the result was 15.4 mg/L (in the hospital with a TDM protocol).

Drawing samples of vancomycin concentration within 30 min before the next scheduled vancomycin administration happened more often in the hospital with a TDM protocol (6.29%/11 of 175 TDMs) than in the hospital without a TDM protocol (4.60%/4 of 87 TDMs), but not with a statistically significant difference (*p* = 0.786). In turn, taking samples of vancomycin concentration during the period of 30–60 min before the administration of a dose happened more often in the hospital without a TDM protocol (5.75%) compared to the hospital with a TDM protocol (3.42%), but also without a statistically significant difference (*p* = 0.579).

Under the title “other” are listed those blood samples after which the next dose of vancomycin was not administered. A total of 158 out of 175 TDMs in the hospital without a TDM protocol and 78 out of 87 TDMs in the hospital with a TDM protocol were not performed within 1 h before vancomycin infusion.

A statistically significant difference (*p* = 0.032) was demonstrated between both hospitals in terms of average concentrations of the first therapeutic drug monitoring of vancomycin 9.72 mg/L and 12.5 mg/L, respectively (Table 6).

In both hospitals, the lowest vancomycin concentration was <0.24 mg/L. In total, 50% of concentration results were in the range of 8.83 mg/L to 15.8 mg/L in the hospital with a TDM protocol. But in the hospital without a TDM protocol, only 25% of vancomycin concentrations were in the range of 9.72 mg/L to 16.1 mg/L after the first TDM. In addition, in the hospital without a TDM protocol, the first vancomycin concentration was below 5.50 mg/L in 25% of patients.

From all the TDMs performed (the hospital without a TDM protocol, *N* = 459, and the hospital with a TDM protocol, *N* = 180), in the hospital with a TDM protocol, most of the vancomycin concentrations (55.56%) were in the therapeutic range 10–20 mg/L (Table 7).

Only in 34.42% of cases the target vancomycin concentration range of 10–20 mg/L was reached in the hospital without a TDM protocol, while in the hospital with a TDM protocol, it was reached in 55.56% of cases (*p* < 0.01). It is noteworthy that in the hospital without a TDM protocol, slightly more than a half (53.38%) of all measurements (459) had vancomycin concentrations below 10 mg/L.

## 4. Discussion

Therapeutic drug monitoring, individualization of dosage regiments, and evaluation of renal function are important aspects that should be taken into consideration at the moment of an initiation of vancomycin therapy for it to be as safe as possible and to achieve a desired therapeutic effect. Inaccurate therapeutic monitoring, dosing of vancomycin, and timing of trough assays may lead to a situation of inadequate concentration of vancomycin, resulting in unsuccessful outcomes and increased risk of toxicity and development of resistant microorganisms [12,13,14].

TDM of vancomycin has been recommended for patients with unstable renal function, long-term vancomycin therapy, and high dose regiments, as well as for special subpopulations and patients with inadequate clinical response [6,13,15]. However, it should be taken into consideration that treatment of a particular patient should be assessed on an individual basis and guided by the clinical situation, recalling that each TDM performed imposes certain costs on the hospital, as well as additional workload for doctors, nurses, and other medical professionals involved in the process.

Administration of a loading dose (25–30 mg/kg based on actual body weight) is recommended for an initiation of vancomycin therapy to achieve the necessary vancomycin blood levels in a faster mode [15]. This recommendation is based on the results of clinical studies evaluating trough serum vancomycin levels after an administration of a loading dose [16]. This strategy in particular could help towards avoiding therapy failure in critically ill patients. Publications indicate that in critically ill patients, vancomycin concentrations often do not reach therapeutic levels (15–20 mg/L) during the first 5 days of vancomycin therapy [17]. For more successful therapy results, vancomycin concentration should reach therapeutic concentration more rapidly [18].

Malaeb et al. (2019) reported that most of the physicians do not administer a loading dose when initiating vancomycin therapy. This could explain why in most of the cases target vancomycin concentration levels cannot be achieved [18]. In our study in the hospital without a TDM protocol, only two patients (1.59%) received a loading dose according to the medical records. It shows that the application of a loading dose is not a routine practice at the initiation of vancomycin therapy. Although in most of the cases in the hospital without a protocol, patients were critically ill, obese, or with augmented renal function, and therefore it would be recommended to initiate vancomycin therapy with an administration of a loading dose. In the hospital with a TDM protocol, a loading dose was administered in 10 (22.73%) patients (*p* < 0.01). In the hospital with a TDM protocol, the clinical pharmacist mandated that the initiation of vancomycin therapy should start with an administration of vancomycin loading dose. Authors, such as Philips et al. (2015), showed the results that, after clinical pharmacist’s intervention, the frequency of administration of a loading dose increased (from 9.43% to 28.27% (*p* = 0.02)) [19].

Administration of a loading dose usually increases concerns about an elevated risk of nephrotoxicity. Therefore, that might be the reasoning behind a clinicians’ decision of starting vancomycin therapy with a routine standard (maintenance) dose. However, Mei et al. (2019) in the systemic review and meta-analysis proved that in patients who received a loading dose as a first dose, the risks of nephrotoxicity or other undesirable effects did not increase [20].

The strategy of maintenance dosing of vancomycin depends on the renal function. In the case of renal impairment, the reduction of a maintenance dose or a prolongation of an interval between the doses would be essential. Such clinical strategies were observed in both hospitals. The most common dosage regiment of vancomycin therapy was 1 g every 12 h. Such practice conforms to the information available in the scientific literature [6].

Results from various studies highlight that appropriate vancomycin therapy is more difficult to be applied for patients with augmented renal function [21]. Augmented renal function is a pathological phenomenon wherein kidneys display increased filtering activity exceeding a normal renal function. Patients in such a condition have GFR > 130 mL/min/1.73 m^2^. As the clearance of vancomycin is faster in this situation, in most cases, vancomycin serum concentrations do not reach therapeutic concentration for these patients. Scientific literature suggests that not only an administration of a loading dose, but also maintenance doses should be higher and administration intervals should be shorter in the respective patients if compared to patients with normal renal function [21,22,23]. While dose adjustments for acute renal failure or renal replacement therapy is a common clinical practice, vancomycin dose adjustments for augmented renal function are not a daily routine [22]. In our study, both hospitals had recommendations for vancomycin dosing adjustments in patients with impaired renal function but not in cases with augmented renal function.

For appropriate interpretation of vancomycin concentration sampling results, it is necessary to investigate the dosing history of a drug (administration times and dosing intervals), clinical response, and therapeutic goals, as well as drug concentration sampling times. For this information to be usable in practice, documenting accurate data is important [24].

Tangedal et al. (2017) identified the most common errors associated with vancomycin therapy, such as failure to document the risk factors for nephrotoxicity, failure to document a proper plan to obtaining the next blood samples of vancomycin concentration, and failure to document a correct timing of blood sampling. The authors stress that such information is essential for a multidisciplinary team, including a clinical pharmacist involved in the evaluation and modification process of vancomycin therapy [24]. The Scottish guidelines also emphasize the importance of correct and complete documentation [25].

Correct time of both vancomycin administration and sampling had been documented only in 18.95% of cases performed in the hospital without a TDM protocol, in contrary to 97.22% of cases in the hospital with a TDM protocol. Failure of recording of both the exact sampling time and the beginning of vancomycin administration in medical records may lead to incorrect interpretation of vancomycin concentration results, which has been reported in several publications [24,25,26].

In addition, due to a retrospective design of this study, and therefore some limitations, information on recorded timing may be biased. Therefore, prospective study would be necessary to be carried out to have unbiased results. Scientific publications indicate that discrepancies between planned and actual timing of sample collection and beginning of vancomycin administration are often observed. Even a mistake of a few minutes both in real life and incorrect documenting can lead to inaccuracies significantly affecting an appropriate evaluation of vancomycin therapy [27].

Correct timing of blood sampling for vancomycin TDM is one of the most challenging steps in the clinical practice. Samples drawn several hours before or immediately after an administration of vancomycin lead to inaccurate interpretation of vancomycin trough concentrations. Situations like this may lead to incorrect adaption of vancomycin therapy [12,24,28].

A vancomycin trough sample should be obtained within 30 min prior to the next dose [7]. In the indicated time interval, 4.60% of the 87 analyses were drawn in the hospital without a TDM protocol compared to 6.29% of the 175 in the hospital with a TDM protocol. There was no statistical significant difference between both hospitals.

It is worth noting that in some cases the times of both clinical manipulations (administration and sampling) were recorded at the same time in both hospitals. There is a standard procedure in the hospital with a TDM protocol requiring a nurse to prepare two trays before approaching a patient, one for materials obtaining a blood sample and the other for all materials and premixed solutions for vancomycin administration, thus stimulating the collection of blood samples at a correct timing. But since there was no possibility to interpret such times of both clinical manipulations (blood sample taken right before or after the administration of vancomycin dose), this group was separated. It is more likely that the prospective design of the study would show a larger number of blood samples taken in the appropriate timeframe in the hospital with a TDM protocol.

The time of trough concentration measurement has been extensively studied in the research literature. The research data confirms that 40%–45% of health care professionals draw blood samples too fast before the next dose of vancomycin, i.e., more than 30 min prior to the next administration. Neeley et al. (2018) reported that the results of blood samples taken at the wrong time do not accurately reflect vancomycin concentrations. As a result, those are not trough concentrations [12]. Damfu et al. (2016) indicated that the majority of blood samples are obtained more than 2 h prior the next administration of vancomycin, emphasizing that obtaining blood samples earlier had higher mean concentrations (22.1 mg/L) when compared to those taken at an appropriate time (15.5 mg/L) (*p* < 0.0001) [29].

The ratio of the 24-h area under the concentration-time curve (AUC24) to the minimum inhibitory concentration (MIC) best characterizes the pharmacodynamic effectiveness of vancomycin. The AUC24/MIC of ≥400 mg*hr/L is associated with improved clinical response and microbiologic eradication of *Staphylococcus aureus* in adult patients. Katip and Oberforfer publication (2021) describes rather similar conclusions about the AUC/MIC ratio in *Enterococcus* spp. infections, where the vancomycin AUC/MIC ≥ 400 mg*h/L ratio had better results in the clinical outcome and eradication of pathogens in comparison to vancomycin the AUC/MIC < 400 mg*h/L ratio [30].

For practical reasons, at least until the current decade, a determination of vancomycin trough concentration has been widely utilized for the control of drug levels and the proper performance of TDM [7]. In the near future, higher AUC/MIC ratio might be applied in practice of clinical pharmacy not only in the Global West, but also in the Global South for more successful treatment of patients with infectious diseases, including *S. aureus* and *Enterococcus* spp. In many clinical settings, concentration measurements of vancomycin might then be used as a surrogate prediction of the AUC/MIC ratio. However, the AUC/MIC ratio is a rather disputable calculation as modest results on patient outcome and considerable variations were described in the systemic review performed by Dalton et al. in 2020 [31,32]. In the 2009 vancomycin dosing guidelines in the USA, it was recommended to maintain serum vancomycin through concentrations in the range of 15–20 mg/L to create the AUC/MIC ratio of 400 to treat severe infections [6]. Other publications also recommend to maintain vancomycin concentrations of 15–20 mg/L in patients with severe infections (bacteraemia, endocarditis, osteomyelitis, meningitis, and nosocomial pneumonia). It has also been stated that vancomycin trough concentration should be >10 mg/L in order to avoid development of resistant strains [6]. That is not only accurate for *Staphylococcus*-caused infections, but also for *Enterococcus*-caused infections. Even precise and homogeneous data are lacking. In the Katip et al. study, results suggested that vancomycin trough concentration of 15–20 mg/L would give the best benefit:risk ratio between the best therapeutic outcomes and the least negative impact on renal function [33].

However, many studies indicate that therapeutic vancomycin serum concentrations during the first days of treatment or even throughout vancomycin therapy is not achieved in the majority of patients [34,35,36]. Achieving sufficient therapeutic concentration of vancomycin in an accelerated manner is associated with better clinical outcomes, especially in critically ill patients [37]. Cardile et al. (2015) reported that the time of discharge and duration of vancomycin therapy may be reduced by achieving the target trough concentration of vancomycin within 5 days after an initiation of vancomycin therapy [17]. Vancomycin concentration of the first TDM was in the therapeutic range (10–20 mg/L) for 52.27% of patients in the hospital with a TDM protocol, compared to only 29.37% in the hospital without a TDM protocol (*p* = 0.011). These results indicate that a more superior choice of the initial dosing regimen of vancomycin is performed in the hospital with a TDM protocol.

After the evaluation of overall results of performed TDMs in both hospitals, it can be concluded that the hospital with a TDM protocol demonstrated better results. In the hospital without a TDM protocol, only in 34.42% of all cases did vancomycin concentration reach the target concentration of 10–20 mg/L. In contrast, in the hospital with a TDM protocol, this number was 55.56% of all performed concentration evaluations. It is important to report that in the hospital without a TDM protocol, slightly more than a half (53.38%) of all samples were with vancomycin concentrations below 10 mg/L. Considering all blood samples with an incorrect timing of collection, the number of samples of vancomycin concentrations in the subtherapeutic range might be even more substantial.

Concentrations up to 5 mg/L or 10 mg/L were considered compliant long before the 2009 US guidelines, and there is a history of this according to current procedures. Such concentrations are currently to be avoided as they increase the bacterial resistance against vancomycin. In the hospital without a TDM protocol, it was observed that medical professionals had many uncertainties about appropriate vancomycin concentration, indicating that, e.g., “vancomycin concentration is adequate” with a concentration of 7.56 mg/L or 4.37 mg/L.

Several studies have indicated an important role of a clinical pharmacist in the improvement of outcomes of vancomycin. Engaging a clinical pharmacist increased the number of patients with a loading dose, more appropriate blood sampling timing, and reaching of therapeutic levels of vancomycin concentration in a more rapid mode. Furthermore, an implementation of a program lead by a clinical pharmacist might improve clinical outcomes and reduce healthcare costs in general [17,37]. The results of this study confirm that the TDM protocol developed by a clinical pharmacist and a subsequent training improved the vancomycin TDM practice compared to the hospital, which did not use such types of collaboration strategy and TDM protocol.

For sustainable results, periodic and regular training of all medical professionals is adequate and necessary. Such educational training would need to include materials and explanation of affecting factors on vancomycin dosing and monitoring, correct administration, timing of blood sampling, target concentrations, dose adjustments after appropriate interpretation of vancomycin concentration results, and, most importantly, renal function. As the details of the evaluation of vancomycin therapy are extremely important, adequately documented notes are important to review and to improve the clinical practice of vancomycin monitoring.

Some limitations should be acknowledged. This was a retrospective study with a rather small number of subjects included, especially in the hospital with a TDM protocol. The hospital without a TDM protocol had significantly more hospitalized patients per year. The hospital with a TDM protocol started to fully monitor vancomycin concentrations after the TDM protocol was approved and implemented in clinical practice in 2018. Other limitations might be that, in the hospital without a TDM protocol, many subjects had to be excluded from the study because of inconsistent and missing data. Therefore, to reach the necessary estimated sample size, we needed to evaluate patients over a longer period of time. In the hospital with a TDM protocol, all data were consistent and chronological. Another study limitation might be highlighted regarding the results of vancomycin through concentration in patients with augmented renal function. Inhouse guidelines on how to precisely proceed with the modifications of vancomycin dosing in patients with augmented renal function were available in neither of the hospitals. The problem with vancomycin dosing in special patient subpopulation groups has been described in literature, mostly leading to lower through concentrations, but evidence is insufficient to make very confident decisions in managing patients with infections [5,23,38,39,40,41].

However, vancomycin is a very effective agent for fighting severe infections if administered appropriately and according to indications and resistance/susceptibility results. An intravenous vancomycin therapy is always associated with certain challenges in clinical practice. Therefore, it is very important to understand and clarify every step of vancomycin therapy to ensure successful results and to avoid undesirable outcomes, including death. Vancomycin-resistant *Staphylococcus aureus* and vancomycin-resistant *Enterococcus* is an increasing problem for healthcare systems due to the emerging resistance issues [42]. Such problems are linked to an initiation of vancomycin therapy in cases when it is not indicated as well as an incorrect dosing regimen of vancomycin if initiated. Furthermore, antibiotic resistance is one of the biggest public health concerns at present. It emerges faster than the development of new antibacterial agents. In addition to infections caused by resistant microorganisms causing an increase in mortality and length of hospitalization, such situations increase healthcare costs all over the globe [10].

Therefore, it is important to clarify the clinical practice of vancomycin administration, including TDM, and improve every detail in its therapy to decrease the risks of resistance and expenditures of the healthcare system. Furthermore, solutions to improve all factors affecting successful vancomycin therapy, including TDM (initial dosing, appropriate timing of blood sampling, target concentration, and their interpretation), need to be implemented to increase the success rate of fighting severe infections where vancomycin is used as the last resource. There have been many efforts to publish harmonized recommendations in countries, addressing the complexity of vancomycin dosing and TDM, but there are still many uncertainties as to how to implement such guidelines effectively [9].

The importance of correct vancomycin dosing, TDM practices, and involvement of clinical pharmacists in improving the outcome of hospitalized patients with infections is very high, but the publications in this field are rather in small numbers. A study discussing the impact of pharmacist interventions in vancomycin therapy was carried out by Komoto et al. and the results were published in 2018. At 30 days after initiation of vancomycin therapy in the intervention group (vancomycin dosing protocol, guided by a pharmacist) resulted in significantly higher survival rate compared to the non-intervention group (82.1% vs. 53.1%) [37]. Such results confirm how important is the process of appropriate vancomycin dosing and TDM practices, preferably lead by a clinical pharmacist as an important a member of a multidisciplinary antimicrobial management team in a hospital [43].

## 5. Conclusions

To ensure the best results of vancomycin therapy in treating severe infections, it is important to comply with all aspects related to vancomycin therapy. One of the important cornerstones includes correct therapeutic drug monitoring. Therefore, individualization of dosage and dosing interval should be adapted more precisely for every patient. Imprecision in therapeutic drug monitoring, dosing of vancomycin, or timing of blood sampling may aggravate the failure of successful treatment of bacterial infections, increased toxicity or, in contrast, selection of resistant bacteria.

The selected hospitals were different in terms of initiation and continuation of vancomycin therapy, including TDM and therapy adjustments during the therapy. More correct therapy was observed in the hospital with a TDM protocol if compared to the hospital without a TDM protocol. Better results were observed in the administration of a loading dose, reaching steady state concentrations faster and necessary therapeutic concentrations more often.

Blood sampling in the correct timeframe before the scheduled vancomycin administration did not show statistically better results in either of the hospitals, but there is an indication that this aspect might be better in the hospital with a TDM protocol.

The results about TDM practice in both hospitals should become a cornerstone for developing evidence-based guidelines, thus being a valuable contribution, for example, to reduce the problem of emerging antimicrobial resistance.

## Figures and Tables

**Table 1:** Demographic and clinical characteristics of patients.

Demographic and Clinical Characteristics	Hospital without a TDM Protocol, *N* = 126	Hospital with a TDM Protocol, *N* = 44
Gender		
Woman, *N* (%) Male, *N* (%)	53 (42.06) 73 (57.94)	16 (36.36) 28 (63.64)
Age, years		
Median (IQR) Min–max	62 (29.5) 19–93	67 (19.75) 35–83
Duration of hospitalization, days		
Median (IQR) Min–max	30 (27.75) 5–233	24 (22.5) 5–103
Weight, kg		
Median (IQR) Min–max Not documented, *N* (%)	75.50 (28.50) 48–140 88 (69.84)	76 (15.00) 70–100 35 (79.55)
Glomerular filtration ratio (GFR), mL/min/1.73 m^2^		
Median (IQR) Min–max	92.70 (79.38) 9.86–285.26	82.5 (67.75) 16–271.26

**Table 2:** Division of six GFR groups depending on GFR value.

	Augmented Renal Function	Normal/Mildly Impaired Renal Function	Moderately Impaired Renal Function		Severe Renal Impairment		***p***
GFR, mL/min/1.73 m^2^	>130	60–130	45–59	30–44	15–29	<15	
GFR groups	Group GFR 1	Group GFR 2	Group GFR 3	Group GFR 4	Group GFR 5	Group GFR 6	
Hospital without a TDM protocol, *N* (%)	29 (23.2)	58 (46.4)	12 (9.6)	11 (8.00)	12 (9.60)	4 (3.20)	0.636
Hospital with a TDM protocol, *N* (%)	8 (18.2)	22 (50.0)	6 (13.6)	2 (4.55)	6 (13.6)	0 (0.00)	

**Table 3:** Dosing regimen of vancomycin until the first TDM based on GFR in the hospital without a TDM protocol.

Dosing Regimen	GFR Groups					
	GFR 1	GFR 2	GFR 3	GFR 4	GFR 5	GFR 6
0.5 g q12h, (%)	0	3.45	0	9.09	8.33	0
1 g q8h, (%)	3.45	5.17	0	0	0	0
1 g q12h, (%)	82.75	82.76	91.67	36.36	25	0
1 g q24h, (%)	6.90	6.90	8.33	54.55	58.34	75
1.5 g q12h, (%)	0	0	0	0	0	0
Other, (%)	6.90	1.72	0	0	8.33	25

**Table 4:** Dosing regimen of vancomycin until the first TDM based on GFR in the hospital with a TDM protocol.

Dosing Regimen	GFR Groups					
	GFR 1	GFR 2	GFR 3	GFR 4	GFR 5	GFR 6
0.5 g q12h, (%)	0	0	0	0	33.33	0
1 g q8h, (%)	12.50	4.55	0	0	0	0
1 g q12h, (%)	62.50	68.18	33.33	100	50	0
1 g q24h, (%)	0	13.64	66.67	0	0	0
1.5 g q12h, (%)	12.50	9.08	0	0	0	0
Other, (%)	12.50	4.55	0	0	16.67	0

**Table 5:** Timing of blood sampling in relation to the next scheduled dose.

Timing of Blood Sampling	Hospital without a TDM Protocol, *N* = 87	Hospital with a TDM Protocol, *N* = 175
Within 30 min prior to next dose, (%)	4.60	6.29
31 min to 1 h prior to next dose, (%)	5.75	3.42
1 h to 2 h prior to next dose, (%)	17.24	14.87
2 h to 3 h prior to next dose, (%)	17.24	21.71
3 h to 10 h prior to next dose, (%)	37.92	14.85
Up to 3 h after administration (%)	9.20	20
Documented as at the same time, (%)	4.60	12
Other, (%)	3.45	6.86

**Table 6:** Values of vancomycin concentration after the first performed TDM in both hospitals.

	Hospital without a TDM Protocol, *N* = 126	Hospital with a TDM Protocol, *N* = 44	*p*
Median [Q1; Q3] Min-Max	9.72 (5.50;16.1) <0.24–160.23	12.5 (8.83;15.8) <0.24–30.2	0.032

**Table 7:** Comparative analysis of vancomycin levels during vancomycin therapy.

Vancomycin Trough Concentration	Hospital without a TDM Protocol, *N* = 459	Hospital with a TDM Protocol, *N* = 180
0–5 mg/L, (%)	18.30	1.11
5–10 mg/L, (%)	35.08	13.89
10–15 mg/L, (%)	23.09	26.67
15–20 mg/L, (%)	11.33	28.89
>20 mg/L, (%)	12.20	29.44

## Data Availability

The data presented in this study are available on request from the corresponding author.
